# Supplementary material for: Modeling glioblastoma heterogeneity as a dynamic network of cell states
Source: Mol Syst Biol. 2021 Sep 16;17(9):e10105. doi: 10.15252/msb.202010105 (PMC8444284; doi:10.15252/msb.202010105)
Supplement: Supplementary file 5 — Source Data for Figure 3 [file MSB-17-e10105-s001.zip › Figure3A_sourcedata/GSEA_3065/hallmarks_state2.GseaPreranked.1623416269049/HALLMARK_PI3K_AKT_MTOR_SIGNALING.html]

Details for gene set HALLMARK\_PI3K\_AKT\_MTOR\_SIGNALING[GSEA]

|  || Dataset | state2 |
| Phenotype | NoPhenotypeAvailable |
| Upregulated in class | na\_pos |
| GeneSet | HALLMARK\_PI3K\_AKT\_MTOR\_SIGNALING |
| Enrichment Score (ES) | 0.37959743 |
| Normalized Enrichment Score (NES) | 1.2444494 |
| Nominal p-value | 0.13839892 |
| FDR q-value | 0.20691699 |
| FWER p-Value | 0.991 |
Table: GSEA Results Summary

  

Fig 1: Enrichment plot: HALLMARK\_PI3K\_AKT\_MTOR\_SIGNALING      
 Profile of the Running ES Score & Positions of GeneSet Members on the Rank Ordered List

  

| PROBE | GENE SYMBOL | GENE\_TITLE | RANK IN GENE LIST | RANK METRIC SCORE | RUNNING ES | CORE ENRICHMENT || 1 | CDK1 |  |  | 18 | 0.577 | 0.0702 | Yes |
| 2 | E2F1 |  |  | 59 | 0.446 | 0.1218 | Yes |
| 3 | CDK4 |  |  | 131 | 0.333 | 0.1558 | Yes |
| 4 | RAC1 |  |  | 166 | 0.301 | 0.1897 | Yes |
| 5 | CFL1 |  |  | 268 | 0.244 | 0.2094 | Yes |
| 6 | PIN1 |  |  | 296 | 0.234 | 0.2358 | Yes |
| 7 | UBE2N |  |  | 325 | 0.224 | 0.2609 | Yes |
| 8 | PFN1 |  |  | 352 | 0.216 | 0.2851 | Yes |
| 9 | YWHAB |  |  | 369 | 0.212 | 0.3099 | Yes |
| 10 | CDK2 |  |  | 382 | 0.208 | 0.3347 | Yes |
| 11 | ARHGDIA |  |  | 546 | 0.173 | 0.3388 | Yes |
| 12 | PPP1CA |  |  | 559 | 0.171 | 0.3589 | Yes |
| 13 | CSNK2B |  |  | 786 | 0.140 | 0.3522 | Yes |
| 14 | UBE2D3 |  |  | 820 | 0.136 | 0.3657 | Yes |
| 15 | EIF4E |  |  | 1038 | 0.116 | 0.3571 | Yes |
| 16 | ARF1 |  |  | 1068 | 0.114 | 0.3682 | Yes |
| 17 | AKT1 |  |  | 1093 | 0.112 | 0.3796 | Yes |
| 18 | ACTR3 |  |  | 1528 | 0.085 | 0.3439 | No |
| 19 | PTPN11 |  |  | 1665 | 0.079 | 0.3392 | No |
| 20 | HRAS |  |  | 1748 | 0.075 | 0.3397 | No |
| 21 | ECSIT |  |  | 1940 | 0.067 | 0.3277 | No |
| 22 | ARPC3 |  |  | 2022 | 0.064 | 0.3270 | No |
| 23 | MAPK9 |  |  | 2139 | 0.060 | 0.3221 | No |
| 24 | AP2M1 |  |  | 2310 | 0.053 | 0.3106 | No |
| 25 | MAPK1 |  |  | 2595 | 0.045 | 0.2858 | No |
| 26 | RPS6KA3 |  |  | 2677 | 0.042 | 0.2825 | No |
| 27 | PRKAA2 |  |  | 2836 | 0.038 | 0.2703 | No |
| 28 | RPS6KA1 |  |  | 2948 | 0.035 | 0.2628 | No |
| 29 | ACTR2 |  |  | 3044 | 0.033 | 0.2568 | No |
| 30 | MAPK8 |  |  | 3153 | 0.031 | 0.2491 | No |
| 31 | GRK2 |  |  | 3192 | 0.030 | 0.2488 | No |
| 32 | PAK4 |  |  | 3199 | 0.030 | 0.2518 | No |
| 33 | RAF1 |  |  | 3447 | 0.025 | 0.2286 | No |
| 34 | MAP2K3 |  |  | 3478 | 0.024 | 0.2284 | No |
| 35 | NFKBIB |  |  | 3488 | 0.024 | 0.2305 | No |
| 36 | ATF1 |  |  | 3548 | 0.023 | 0.2270 | No |
| 37 | PRKAG1 |  |  | 3746 | 0.020 | 0.2084 | No |
| 38 | TRAF2 |  |  | 3754 | 0.020 | 0.2101 | No |
| 39 | MYD88 |  |  | 3818 | 0.019 | 0.2057 | No |
| 40 | GRB2 |  |  | 3991 | 0.016 | 0.1893 | No |
| 41 | CDKN1B |  |  | 4019 | 0.015 | 0.1883 | No |
| 42 | RIPK1 |  |  | 4140 | 0.013 | 0.1771 | No |
| 43 | AKT1S1 |  |  | 4327 | 0.010 | 0.1585 | No |
| 44 | MAPKAP1 |  |  | 4448 | 0.008 | 0.1467 | No |
| 45 | RALB |  |  | 4701 | 0.004 | 0.1203 | No |
| 46 | PRKAR2A |  |  | 4807 | 0.003 | 0.1094 | No |
| 47 | PLCB1 |  |  | 5022 | -0.000 | 0.0865 | No |
| 48 | PLA2G12A |  |  | 5176 | -0.003 | 0.0706 | No |
| 49 | CAB39 |  |  | 5216 | -0.004 | 0.0668 | No |
| 50 | MAP3K7 |  |  | 5296 | -0.005 | 0.0590 | No |
| 51 | IRAK4 |  |  | 5793 | -0.013 | 0.0076 | No |
| 52 | DUSP3 |  |  | 5805 | -0.013 | 0.0081 | No |
| 53 | VAV3 |  |  | 5899 | -0.015 | -0.0000 | No |
| 54 | MAP2K6 |  |  | 5956 | -0.016 | -0.0040 | No |
| 55 | PIKFYVE |  |  | 6052 | -0.017 | -0.0120 | No |
| 56 | THEM4 |  |  | 6187 | -0.020 | -0.0239 | No |
| 57 | PPP2R1B |  |  | 6250 | -0.021 | -0.0279 | No |
| 58 | ACACA |  |  | 6253 | -0.021 | -0.0255 | No |
| 59 | GSK3B |  |  | 6331 | -0.022 | -0.0310 | No |
| 60 | SMAD2 |  |  | 6404 | -0.024 | -0.0357 | No |
| 61 | DDIT3 |  |  | 6433 | -0.024 | -0.0357 | No |
| 62 | PLCG1 |  |  | 6504 | -0.025 | -0.0400 | No |
| 63 | TBK1 |  |  | 6762 | -0.031 | -0.0636 | No |
| 64 | TIAM1 |  |  | 6948 | -0.035 | -0.0790 | No |
| 65 | MKNK2 |  |  | 7371 | -0.046 | -0.1183 | No |
| 66 | NCK1 |  |  | 7576 | -0.053 | -0.1335 | No |
| 67 | RIT1 |  |  | 7880 | -0.062 | -0.1581 | No |
| 68 | EGFR |  |  | 8094 | -0.071 | -0.1720 | No |
| 69 | PTEN |  |  | 8202 | -0.077 | -0.1738 | No |
| 70 | TSC2 |  |  | 8259 | -0.080 | -0.1697 | No |
| 71 | CLTC |  |  | 8571 | -0.101 | -0.1903 | No |
| 72 | SQSTM1 |  |  | 8790 | -0.121 | -0.1985 | No |
| 73 | PIK3R3 |  |  | 8840 | -0.127 | -0.1878 | No |
| 74 | ITPR2 |  |  | 8925 | -0.138 | -0.1796 | No |
| 75 | TNFRSF1A |  |  | 8962 | -0.143 | -0.1655 | No |
| 76 | ADCY2 |  |  | 9004 | -0.149 | -0.1512 | No |
| 77 | MAPK10 |  |  | 9041 | -0.156 | -0.1355 | No |
| 78 | STAT2 |  |  | 9134 | -0.174 | -0.1236 | No |
| 79 | CDKN1A |  |  | 9301 | -0.230 | -0.1126 | No |
| 80 | CALR |  |  | 9310 | -0.237 | -0.0838 | No |
| 81 | CXCR4 |  |  | 9328 | -0.251 | -0.0543 | No |
| 82 | HSP90B1 |  |  | 9334 | -0.257 | -0.0227 | No |
| 83 | SLC2A1 |  |  | 9349 | -0.272 | 0.0097 | No |
Table: GSEA details [plain text format]

  

Fig 2: HALLMARK\_PI3K\_AKT\_MTOR\_SIGNALING: Random ES distribution      
 Gene set null distribution of ES for **HALLMARK\_PI3K\_AKT\_MTOR\_SIGNALING**
